# Supplementary figures and images for: phyloFlash: Rapid Small-Subunit rRNA Profiling and Targeted Assembly from Metagenomes
Source: mSystems. 2020 Oct 27;5(5):e00920-20. doi: 10.1128/mSystems.00920-20 (PMC7593591; doi:10.1128/mSystems.00920-20)

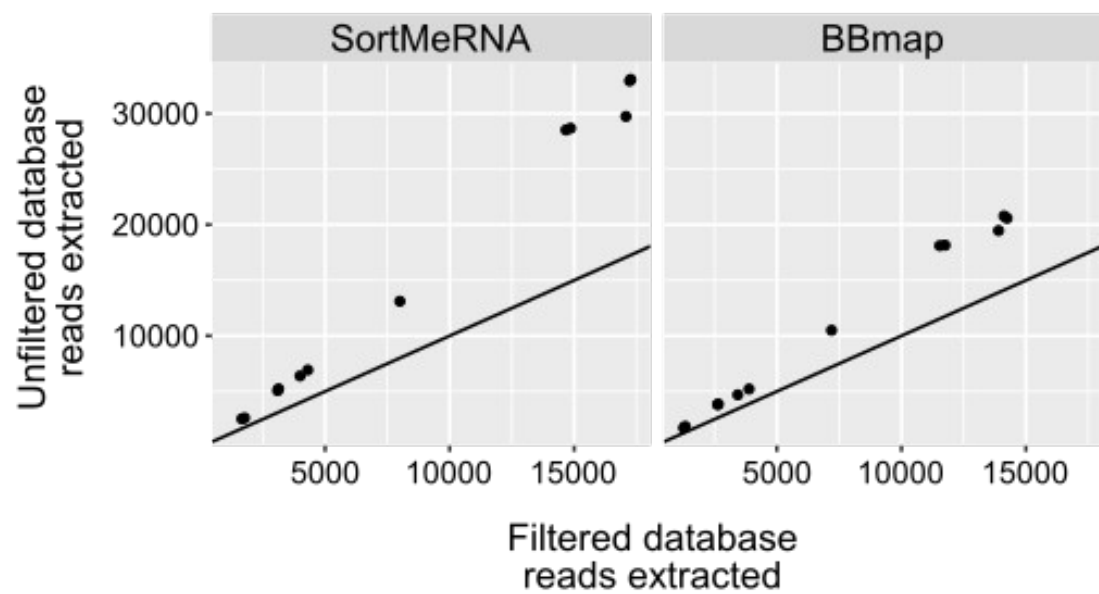

Supplement: FIG S1 [file mSystems.00920-20-sf001.pdf]

Comparison of assembled reads per library extracted by SortMeRNA vs BBmap

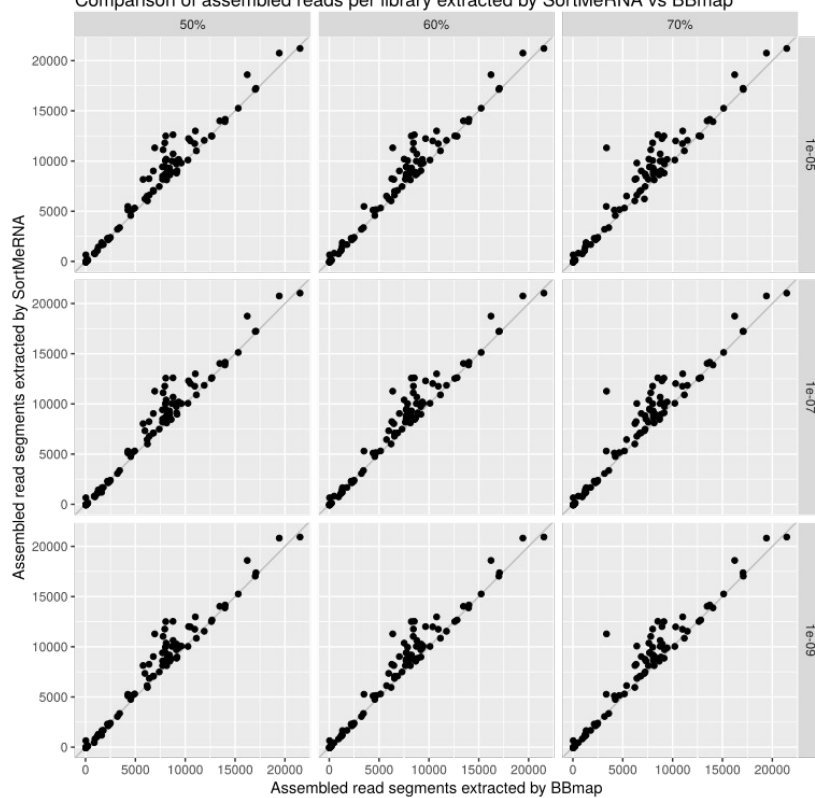

Supplement: FIG S3 [file mSystems.00920-20-sf003.pdf]

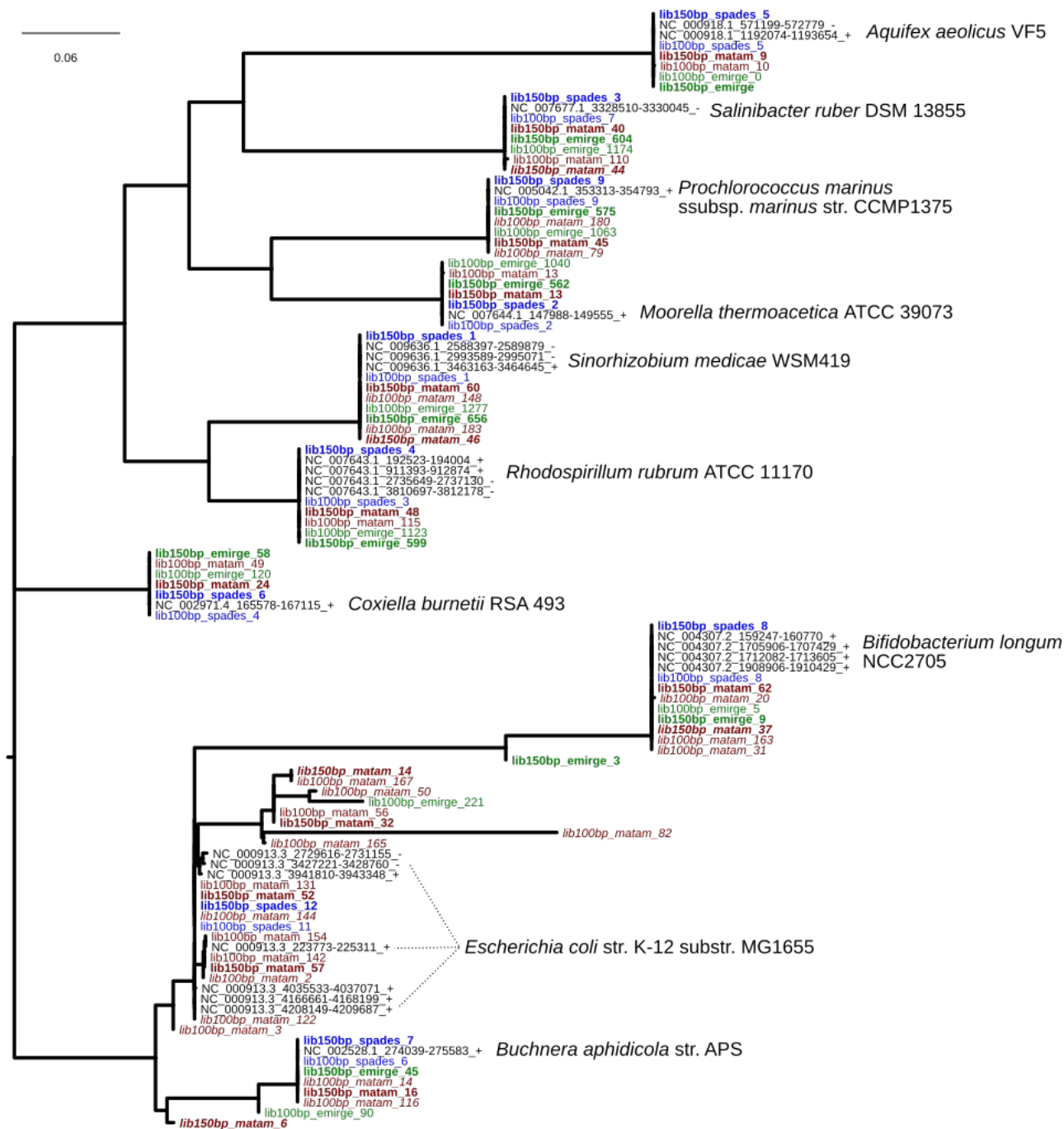

Supplement: FIG S4 [file mSystems.00920-20-sf004.pdf]

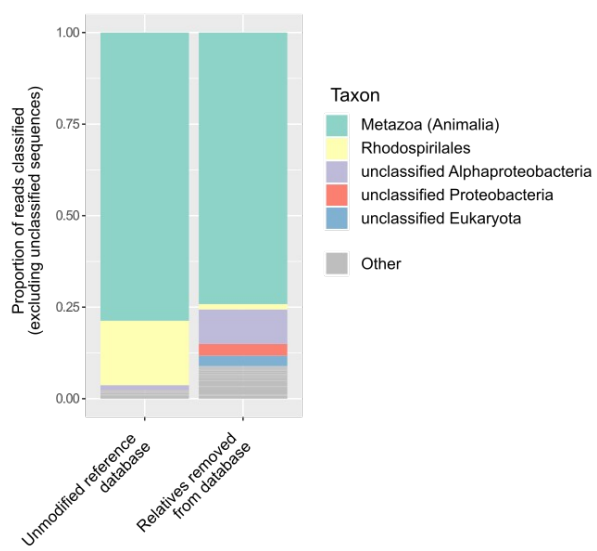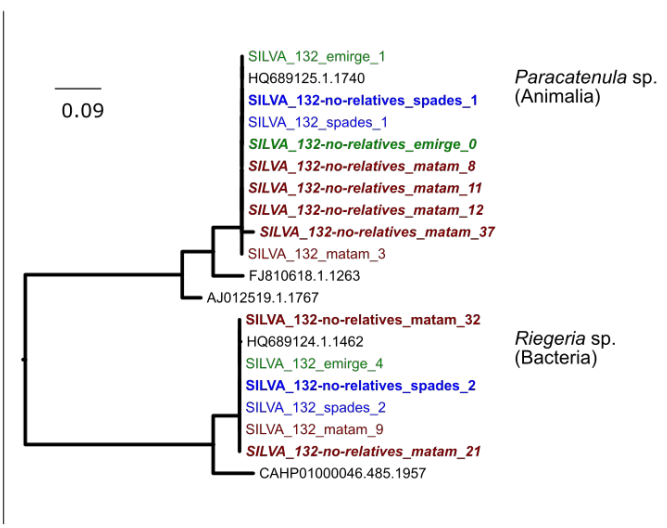

Supplement: FIG S6 [file mSystems.00920-20-sf006.pdf]
