# Supplementary material for: phyloFlash: Rapid Small-Subunit rRNA Profiling and Targeted Assembly from Metagenomes
Source: mSystems. 2020 Oct 27;5(5):e00920-20. doi: 10.1128/mSystems.00920-20 (PMC7593591; doi:10.1128/mSystems.00920-20)
Supplement: TABLE S1 [file mSystems.00920-20-st001.docx]

**Supplementary Table 1**

| **Library profile** | **Software** | **Setting** | **Reads extracted** | | | **Selectivity** | **Sensitivity** | **Timing (min)** |
| --- | --- | --- | --- | --- | --- | --- | --- | --- |
|  |  |  | **Non-SSU rRNA** | **SSU rRNA** | **Total** |  |  |  |
| HiSeq2000, 100 bp PE | BBmap | Identity > 50% | 4349 | 19473 | 23822 | 0.817 | 0.963 | 2.35 |
| HiSeq2000, 100 bp PE | BBmap | Identity > 60% | 3516 | 19420 | 22936 | 0.847 | 0.961 | 2.40 |
| HiSeq2000, 100 bp PE | BBmap | Identity > 70% | 1747 | 19021 | 20768 | 0.916 | 0.941 | 2.75 |
| HiSeq2000, 100 bp PE | SortMeRNA | E-value < 10^-5^ | 4126 | 19245 | 23371 | 0.823 | 0.952 | 11.78 |
| HiSeq2000, 100 bp PE | SortMeRNA | E-value < 10^-7^ | 3222 | 19143 | 22365 | 0.856 | 0.947 | 9.40 |
| HiSeq2000, 100 bp PE | SortMeRNA | E-value < 10^-9^ | 2392 | 19035 | 21427 | 0.888 | 0.942 | 9.02 |
| HiSeq2500, 150 bp PE | BBmap | Identity > 50% | 2093 | 13246 | 15339 | 0.864 | 0.958 | 1.38 |
| HiSeq2500, 150 bp PE | BBmap | Identity > 60% | 1489 | 13183 | 14672 | 0.899 | 0.953 | 1.38 |
| HiSeq2500, 150 bp PE | BBmap | Identity > 70% | 650 | 12868 | 13518 | 0.952 | 0.931 | 1.43 |
| HiSeq2500, 150 bp PE | SortMeRNA | E-value < 10^-5^ | 4902 | 13368 | 18270 | 0.732 | 0.967 | 13.7 |
| HiSeq2500, 150 bp PE | SortMeRNA | E-value < 10^-7^ | 3809 | 13331 | 17140 | 0.778 | 0.964 | 13.4 |
| HiSeq2500, 150 bp PE | SortMeRNA | E-value < 10^-9^ | 2990 | 13288 | 16278 | 0.816 | 0.961 | 13.4 |
