# Supplementary material for: phyloFlash: Rapid Small-Subunit rRNA Profiling and Targeted Assembly from Metagenomes
Source: mSystems. 2020 Oct 27;5(5):e00920-20. doi: 10.1128/mSystems.00920-20 (PMC7593591; doi:10.1128/mSystems.00920-20)
Supplement: FIG S2 [file mSystems.00920-20-sf002.pdf]

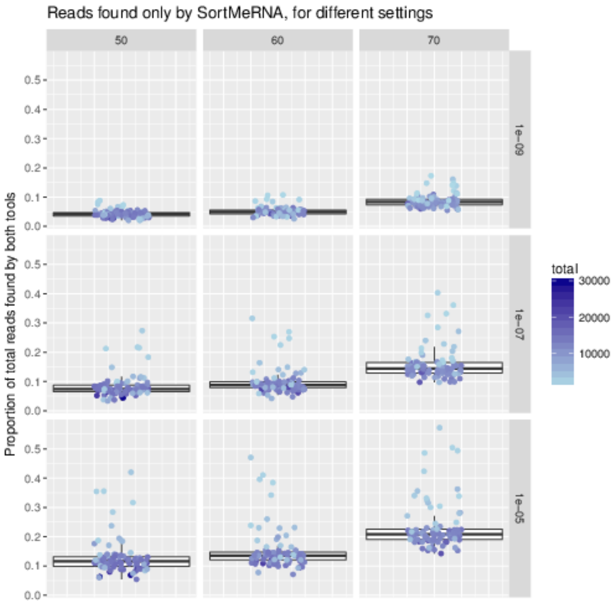
Reads found only by SortMeRNA, for different settings
50
60
70
1e−09
1e−07
1e−05
Proportion of total reads found by both tools
0.0
0.1
0.2
0.3
0.4
0.5
total
30000
20000
10000

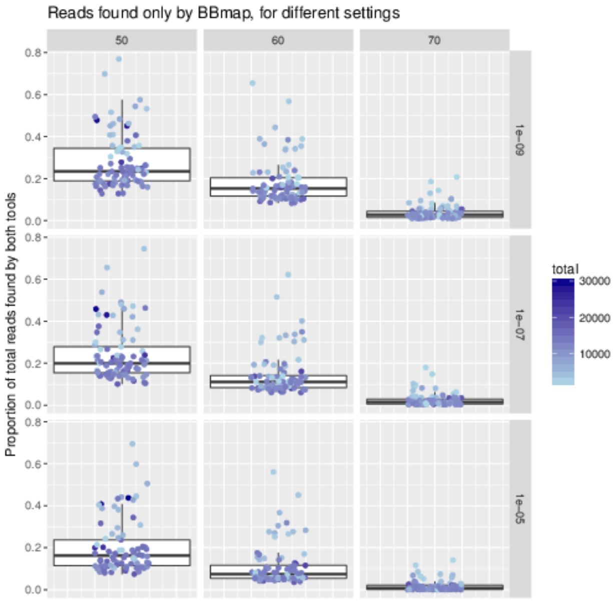
Reads found only by BBmap, for different settings
50
60
70
1e−09
1e−07
1e−05
Proportion of total reads found by both tools
0.0
0.2
0.4
0.6
0.8
total
30000
20000
10000
