# Supplementary material for: phyloFlash: Rapid Small-Subunit rRNA Profiling and Targeted Assembly from Metagenomes
Source: mSystems. 2020 Oct 27;5(5):e00920-20. doi: 10.1128/mSystems.00920-20 (PMC7593591; doi:10.1128/mSystems.00920-20)
Supplement: TABLE S2 [file mSystems.00920-20-st002.docx]

**Supplementary Table 2**

| **SortMeRNA E-value** | **BBmap min. %id** | **Min.** | **1st Quartile** | **Median** | **Mean** | **3rd Quartile** | **Max.** |
| --- | --- | --- | --- | --- | --- | --- | --- |
| 10^-5^ | 50 | 0 | 0.840 | 0.934 | 0.882 | 0.988 | 1.11 |
| 10^-5^ | 60 | 0 | 0.816 | 0.923 | 0.869 | 0.991 | 1.11 |
| 10^-5^ | 70 | 0 | 0.802 | 0.918 | 0.859 | 0.992 | 1.13 |
| 10^-7^ | 50 | 0 | 0.840 | 0.933 | 0.895 | 0.998 | 1.26 |
| 10^-7^ | 60 | 0 | 0.822 | 0.922 | 0.881 | 0.997 | 1.12 |
| 10^-7^ | 70 | 0 | 0.810 | 0.926 | 0.870 | 0.999 | 1.12 |
| 10^-9^ | 50 | 0 | 0.853 | 0.934 | 0.906 | 1.000 | 1.63 |
| 10^-9^ | 60 | 0 | 0.843 | 0.928 | 0.888 | 0.998 | 1.04 |
| 10^-9^ | 70 | 0 | 0.816 | 0.928 | 0.878 | 1.00 | 1.04 |
