# Supplementary material for: phyloFlash: Rapid Small-Subunit rRNA Profiling and Targeted Assembly from Metagenomes
Source: mSystems. 2020 Oct 27;5(5):e00920-20. doi: 10.1128/mSystems.00920-20 (PMC7593591; doi:10.1128/mSystems.00920-20)
Supplement: FIG S5 [file mSystems.00920-20-sf005.pdf]

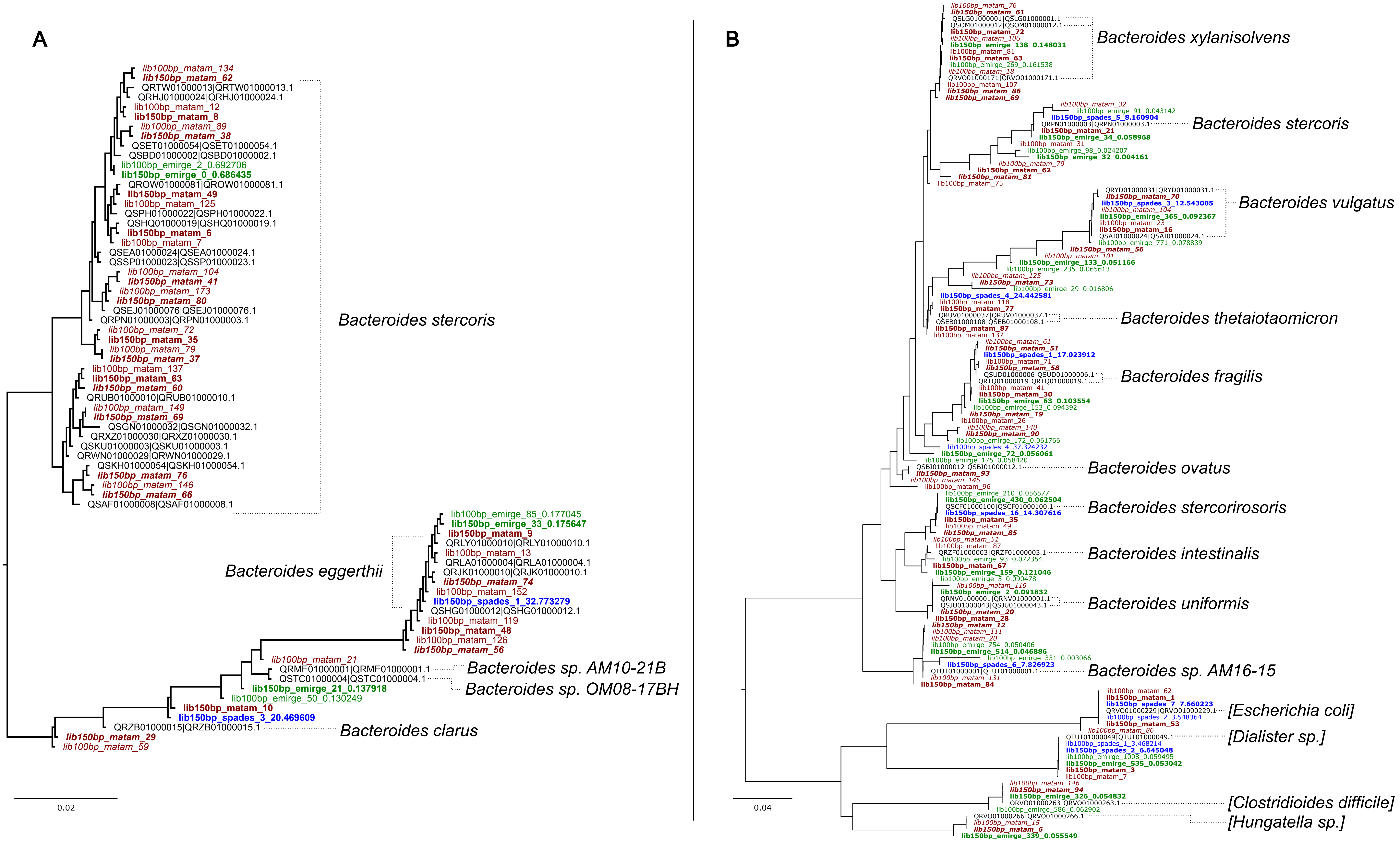
A
lib100bp_matam_134
lib150bp_matam_62
QRTW01000013|QRTW01000013.1
QRHJ01000024|QRHJ01000024.1
lib100bp_matam_12
lib150bp_matam_8
lib100bp_matam_89
lib150bp_matam_38
QSET01000054|QSET01000054.1
QSBD01000002|QSBD01000002.1
lib100bp_emirge_2_0.692706
lib150bp_emirge_0_0.686435
QROW01000081|QROW01000081.1
lib150bp_matam_49
lib100bp_matam_125
QSPH01000022|QSPH01000022.1
QSHQ01000019|QSHQ01000019.1
lib150bp_matam_6
lib100bp_matam_7
QSEA01000024|QSEA01000024.1
QSSP01000023|QSSP01000023.1
lib100bp_matam_104
lib150bp_matam_41
lib100bp_matam_173
lib150bp_matam_80
QSEJ01000076|QSEJ01000076.1
QRPN01000003|QRPN01000003.1
lib100bp_matam_72
lib150bp_matam_35
lib100bp_matam_79
lib150bp_matam_37
lib100bp_matam_137
lib150bp_matam_63
lib150bp_matam_60
QRUB01000010|QRUB01000010.1
lib100bp_matam_149
lib150bp_matam_69
QSGN01000032|QSGN01000032.1
QRXZ01000030|QRXZ01000030.1
QSKU01000003|QSKU01000003.1
QRWN01000029|QRWN01000029.1
QSKH01000054|QSKH01000054.1
lib150bp_matam_76
lib100bp_matam_146
lib150bp_matam_66
QSAF01000008|QSAF01000008.1
Bacteroides stercoris
lib100bp_emirge_85_0.177045
lib150bp_emirge_33_0.175647
lib150bp_matam_9
QRLY01000010|QRLY01000010.1
lib100bp_matam_13
QRLA01000004|QRLA01000004.1
QRJK01000010|QRJK01000010.1
lib150bp_matam_74
lib100bp_matam_152
lib150bp_spades_1_32.773279
QSHG01000012|QSHG01000012.1
lib100bp_matam_119
lib150bp_matam_48
lib100bp_matam_126
lib150bp_matam_56
Bacteroides eggerthii
lib100bp_matam_21
QRME01000001|QRME01000001.1
QSTC01000004|QSTC01000004.1
Bacteroides sp. AM10-21B
Bacteroides sp. OM08-17BH
lib150bp_emirge_21_0.137918
lib100bp_emirge_50_0.130249
lib150bp_matam_10
lib150bp_spades_3_20.469609
QRZB01000015|QRZB01000015.1
Bacteroides clarus
lib150bp_matam_29
lib100bp_matam_59
0.02
B
lib100bp_matam_76
lib150bp_matam_61
QSLG01000001|QSLG01000001.1
QSOM01000012|QSOM01000012.1
lib150bp_matam_72
lib100bp_matam_106
lib150bp_emirge_138_0.148031
lib100bp_matam_81
lib150bp_matam_63
lib100bp_emirge_269_0.161538
lib100bp_matam_18
QRVO01000171|QRVO01000171.1
Bacteroides xylanisolvens
lib100bp_matam_107
lib150bp_matam_86
lib150bp_matam_69
lib100bp_matam_32
lib100bp_emirge_91_0.043142
lib150bp_spades_5_8.160904
QRPN01000003|QRPN01000003.1
Bacteroides stercoris
lib150bp_matam_21
lib150bp_emirge_34_0.058968
lib100bp_matam_31
lib100bp_emirge_98_0.024207
lib150bp_emirge_32_0.004161
lib100bp_matam_79
lib150bp_matam_62
lib150bp_matam_81
lib100bp_matam_75
QRYD01000031|QRYD01000031.1
lib150bp_matam_70
lib150bp_spades_3_12.543005
Bacteroides vulgatus
lib100bp_matam_104
lib150bp_emirge_365_0.092367
lib100bp_matam_23
lib150bp_matam_16
QSAI01000024|QSAI01000024.1
lib100bp_emirge_771_0.078839
lib150bp_matam_56
lib100bp_matam_101
lib150bp_emirge_133_0.051166
lib100bp_emirge_235_0.065613
lib100bp_matam_125
lib150bp_matam_73
lib100bp_emirge_29_0.016806
lib150bp_spades_4_24.442581
lib100bp_matam_118
lib150bp_matam_77
QRUV01000037|QRUV01000037.1
QSEB01000108|QSEB01000108.1
Bacteroides thetaiotaomicron
lib150bp_matam_87
lib100bp_matam_137
lib100bp_matam_61
lib150bp_matam_51
lib150bp_spades_1_17.023912
lib100bp_matam_71
lib150bp_matam_58
QSUD01000006|QSUD01000006.1
QRTQ01000019|QRTQ01000019.1
Bacteroides fragilis
lib100bp_matam_41
lib150bp_matam_30
lib150bp_emirge_63_0.103554
lib100bp_emirge_153_0.094392
lib150bp_matam_19
lib100bp_matam_26
lib100bp_matam_140
lib150bp_matam_90
lib100bp_emirge_172_0.061766
lib100bp_spades_4_37.324232
lib150bp_emirge_72_0.056061
lib100bp_emirge_175_0.058420
QSBI01000012|QSBI01000012.1
Bacteroides ovatus
lib150bp_matam_93
lib100bp_matam_145
lib100bp_matam_96
lib100bp_emirge_210_0.056577
lib150bp_emirge_430_0.062504
QSCF01000100|QSCF01000100.1
Bacteroides stercorirosoris
lib150bp_spades_16_14.307616
lib150bp_matam_35
lib100bp_matam_49
lib150bp_matam_85
lib100bp_matam_51
lib100bp_matam_87
QRZF01000003|QRZF01000003.1
Bacteroides intestinalis
lib100bp_emirge_93_0.072354
lib150bp_matam_67
lib150bp_emirge_159_0.121046
lib100bp_emirge_5_0.090478
lib100bp_matam_119
lib150bp_emirge_2_0.091832
QRNV01000001|QRNV01000001.1
QSJU01000043|QSJU01000043.1
Bacteroides uniformis
lib150bp_matam_20
lib150bp_matam_28
lib150bp_matam_12
lib100bp_matam_111
lib100bp_matam_20
lib100bp_emirge_754_0.050406
lib150bp_emirge_514_0.046886
lib100bp_emirge_331_0.003066
lib150bp_spades_6_7.826923
QTUT01000001|QTUT01000001.1
Bacteroides sp. AM16-15
lib100bp_matam_131
lib150bp_matam_84
lib100bp_matam_62
lib150bp_matam_1
lib150bp_spades_7_7.660223
QRVO01000229|QRVO01000229.1
[Escherichia coli]
lib100bp_spades_2_3.548364
lib150bp_matam_53
lib100bp_matam_86
QTUT01000049|QTUT01000049.1
[Dialister sp.]
lib100bp_spades_1_3.468214
lib150bp_spades_2_6.645048
lib100bp_emirge_1008_0.059495
lib150bp_emirge_535_0.053042
lib150bp_matam_3
lib100bp_matam_7
lib100bp_matam_146
lib150bp_matam_94
lib150bp_emirge_326_0.054832
QRVO01000263|QRVO01000263.1
[Clostridioides difficile]
lib100bp_emirge_586_0.062902
QRVO01000266|QRVO01000266.1
[Hungatella sp.]
lib100bp_matam_15
lib150bp_matam_6
lib150bp_emirge_339_0.055549
0.04
