# Supplementary material for: phyloFlash: Rapid Small-Subunit rRNA Profiling and Targeted Assembly from Metagenomes
Source: mSystems. 2020 Oct 27;5(5):e00920-20. doi: 10.1128/mSystems.00920-20 (PMC7593591; doi:10.1128/mSystems.00920-20)
Supplement: DATA SET S2 [file mSystems.00920-20-sd002.docx]

**Supplementary Information.** Code to reproduce phyloFlash usage examples

The code presented (for Unix/Linux systems) assumes that phyloFlash scripts are accessible from the path, dependencies have already been installed, and that the user is not behind a proxy server. Each analysis should be run in a separate folder. Detailed installation and usage instructions are provided in the online manual: <https://hrgv.github.io/phyloFlash/>

**Download and set up phyloFlash database**

phyloFlash_makedb.pl --remote

**Low-diversity metagenome**

ENA_phyloFlash.pl --acc ERR2931548 –phyloFlash="--everything --readlength 150" --CPUs 12

**Comparison of multiple metagenome samples**

## Download reads from ENA by run accession number

## and run phyloFlash with a single wrapper script

for ACC in SRR5248183 SRR5413717 SRR5418742 SRR5420142 SRR5420603 SRR6232151

do

ENA_phyloFlash.pl --acc $ACC --phyloFlash="--everything" --CPUs 12

done

## Compare samples and plot heatmap in PDF format

phyloFlash_compare.pl --task heatmap --allzip --level 4 \

--out phyloFlash_gutless_oligochaete_comparison --outfmt pdf

**Connecting SSU rRNA sequences to other contigs in metagenome assembly**

LIB=ERR594304 # Library name prefix

READF=ERR594304_1.fastq.gz # Forward read file

READR=ERR594304_2.fastq.gz # Reverse read file

#run phyloFlash on raw reads

#phyloFlash.pl -lib ${LIB}_pf -read1 $READF -read2 $READF \

-everything -CPUs 12 -readlength 100

#quality and adapter trim reads

mkdir trimmed

cd trimmed

bbduk.sh ref=adapters.fa ktrim=l minlength=36 mink=11 hdist=1 \

in=../$READF in2=../$READR out=${LIB}_ktriml.fq.gz

bbduk.sh ref=adapters.fa ktrim=r trimq=2 qtrim=rl minlength=36 mink=11 hdist=1 \

in=${LIB}_ktriml.fq.gz interleaved=t out=${LIB}_q2_ktrimmed.fq.gz

cd ..

#kmer filter reads

mkdir filtered/

cd filtered

bbnorm.sh -Xmx200g in=../trimmed/${LIB}_q2_ktrimmed.fq.gz \

lowbindepth=3 highbindepth=6 outhigh=${LIB}_q2_ktrimmed_k31higher6.fq.gz \

passes=1 threads=24 interleaved=t

cd ..

#assemble with megahit

as=${LIB}_all_kfilt_MH_21_93; #rename

mkdir assemblies -p

cd assemblies

megahit --k-min 21 --k-max 93 --k-step 20 -m 0.9 -t 48 \

--out-prefix $as \

--12 ../filtered/${LIB}_q2_ktrimmed_k31higher6.fq.gz \

-o $as

megahit_toolkit contig2fastg 93 ./${as}/intermediate_contigs/k93.contigs.fa > ./${as}/${as}.fastg

cd ..

#fastgfish megahit assembly

mkdir bins -p

cd bins

ln -s ../assemblies/${as}/intermediate_contigs/k93.contigs.fa \

./${as}.k93.contigs.fasta

ln -s ../assemblies/${as}/${as}.fastg .

phyloFlash_fastgFishing.pl --fasta ${as}.k93.contigs.fasta \

--fastg ${as}.fastg \

--out ${as}_fastgbin --compare-zip \

../${LIB}_pf.phyloFlash.tar.gz \

--assembler megahit --outfasta \

--min-SSU-frac 0.6

cd ../assemblies

#assemble with spades

as_sp=${LIB}_all_kfilt_SPm_21_93; #rename

spades.py --meta -k 21,33,55,77,93 -m 880 -t 48 \

-o $as_sp --12 ../filtered/${LIB}_q2_ktrimmed_k31higher6.fq.gz

#fastgfish spades assembly

cd ../bins;

ln -s ../assemblies/${as_sp}/scaffolds.fasta ./${as_sp}.scaffolds.fasta

ln -s ../assemblies/${as_sp}/assembly_graph.fastg \

./${as_sp}.assembly_graph.fastg

ln -s ../assemblies/${as_sp}/scaffolds.paths \

./${as_sp}.scaffolds.paths

phyloFlash_fastgFishing.pl --fasta ${as_sp}.scaffolds.fasta \

--fastg ${as_sp}.assembly_graph.fastg \

--paths ${as_sp}.scaffolds.paths \

--out ${as_sp}_fastgbin \

--compare-zip ../${LIB}_pf.phyloFlash.tar.gz \

--assembler spades --outfasta --min-SSU-frac 0.6
